# An Acquired Factor VIII Inhibitor in a Patient with HIV and HCV: A Case Presentation and Literature Review

**DOI:** 10.1155/2013/628513

**Published:** 2013-09-30

**Authors:** S. B. Zeichner, A. Harris, G. Turner, M. Francavilla, J. Lutzky

**Affiliations:** ^1^Department of Internal Medicine, Mount Sinai Medical Center, Miami Beach, FL 33140, USA; ^2^Department of Radiology, Mount Sinai Medical Center, Miami Beach, FL 33140, USA; ^3^Division of Hematology/Oncology, Mount Sinai Medical Center, Miami Beach, FL 33140, USA

## Abstract

*Introduction*. Despite its low incidence, acquired factor VIII inhibitor is the most common autoantibody affecting the clotting cascade. The exact mechanism of acquisition remains unclear, but postpartum patients, those with autoimmune conditions or malignancies, and those with exposure to particular drugs appear most susceptible. There have been several case reports describing acquired FVIII inhibitors in patients receiving interferon alpha for HCV treatment and in patients being treated for HIV. To our knowledge, this is the first case of a patient with HCV and HIV who was not actively receiving treatment for either condition. *Case Presentation*. A 57-year-old Caucasian male with a history of HIV and HCV was admitted to our hospital for a several day history of progressively worsening right thigh bruising and generalized weakness. CTA of the abdominal arteries revealed large bilateral retroperitoneal hematomas. Laboratory studies revealed the presence of a high titer FVIII inhibitor. *Conclusion*. Our case of a very rare condition highlights the importance of recognizing and understanding the diagnosis of acquired FVIII inhibitor. Laboratory research and clinical data on the role of newer agents are needed in order to better characterize disease pathogenesis, disease associations, genetic markers, and optimal disease management.

## 1. Introduction

Despite its low incidence of 1.3 to 1.5 patients per million per year [1, 2], acquired factor VIII (FVIII) inhibitor, or acquired hemophilia A, is the most common autoantibody affecting the clotting cascade [3–5]. Incidence increases with age, with a median age of onset of 74 years [2, 6]. The exact mechanism of acquisition remains unclear, and the most common disease associations are idiopathic (64%; [7]), autoimmune conditions (16%; [8–11]), malignancies (12%; [12–17]), pregnancy (8%; [3, 4, 18, 19]), and exposure to particular drugs (5–10%; [5, 20]). The severity of the bleeding, response to treatment, and overall prognosis are heterogeneous with a mortality rate of 8–22% [3, 21, 22].

There have been several case reports describing acquired FVIII inhibitors in patients receiving interferon alpha for hepatitis C virus (HCV) treatment [23–27] and in immune reconstitution inflammatory syndrome (IRIS) in patients being treated for human immunodeficiency virus/acquired immunodeficiency syndrome (HIV/AIDS; [28–32]). To our knowledge, this is the first case of a patient with HCV and HIV who was not actively receiving treatment for either condition.

## 2. Case Presentation

**Figure 1:**
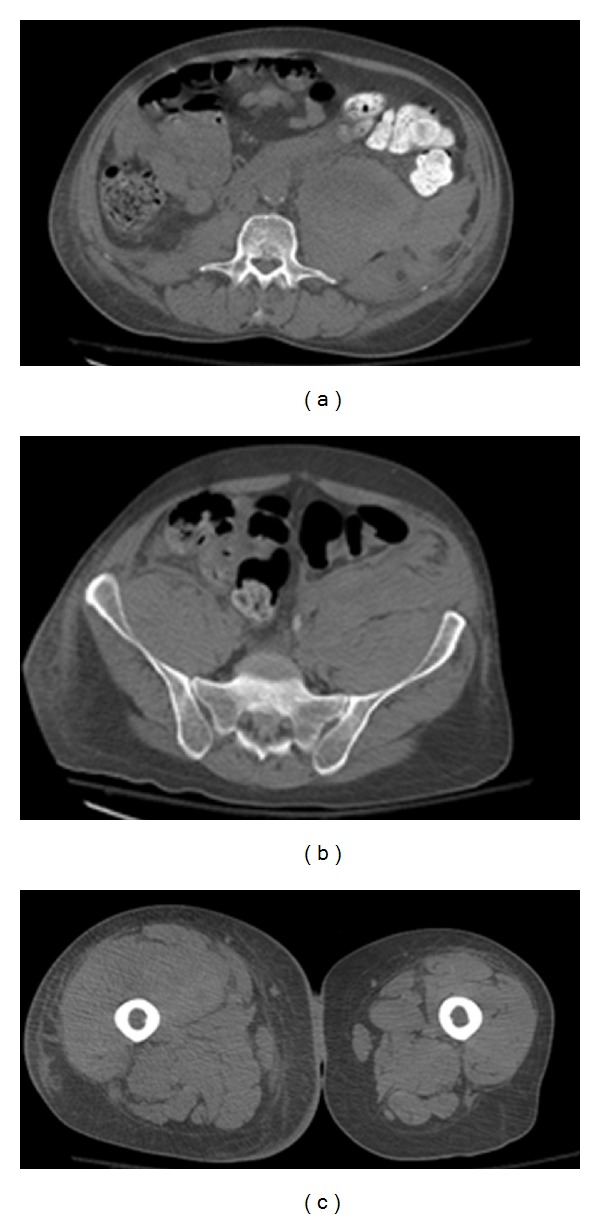
On admission, computed tomography angiogram (CTA) of the abdominal arteries without the use of intravenous contrast revealed large hyperdense retroperitoneal hematomas expanding both psoas muscles, left more than right, that extend into the iliopsoas muscles, and right quadriceps femoris musculature.

**Figure 2:**
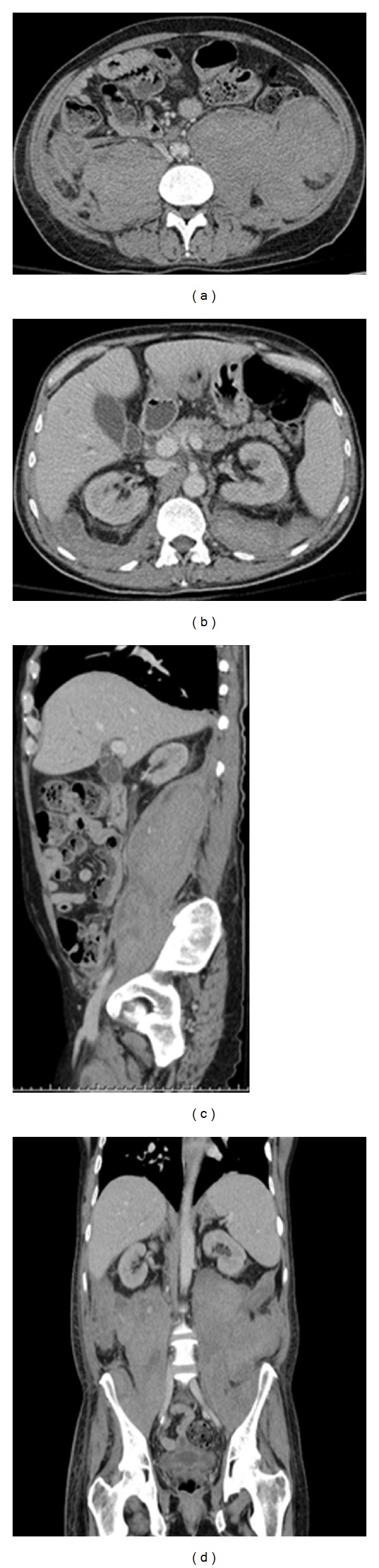
Repeat computed tomography of the abdomen and pelvis demonstrating hematoma stability. Better seen are mixed-aged bilateral retroperitoneal hematomas in the posterior pararenal spaces displacing both kidneys anteriorly with marked mass effect on peritoneal structures.

A 57-year-old Caucasian male was seen in our emergency department for a several day history of progressively worsening right thigh bruising and generalized weakness. His past medical history was notable for HIV (diagnosed ten years before; not on highly active antiretroviral treatment-HAART), HCV (diagnosed ten years before; never treated), end stage renal disease (etiology unclear; on hemodialysis for the previous five months), non-Hodgkin's lymphoma (NHL; diagnosed seven years before; underwent treatment with radiation and chemotherapy, rituximab, cyclophosphamide, vincristine, doxorubicin, and prednisone-R-CHOP; had a complete response and has been in remission ever since), diabetes type 2 (noninsulin dependent), nicotine abuse (48-year history), and peripheral neuropathy. Surgical history was notable for the placement of a left arm arteriovenous (AV) fistula. Patient's presenting medications, none of which were started in the previous month, included zolpidem, pregabalin, duloxetine, sucralfate, pantoprazole, glipizide, and vitamins. On examination, the patient was afebrile, tachycardic at 118 beats per minute, normotensive, and was breathing comfortably on room air. Physical exam was notable for an anxious appearing male with bilateral conjunctiva pallor. He had a left arm fistula with a thrill and a large right thigh ecchymosis with associated edema. Laboratory studies were notable for hemoglobin 7.2 g/dL, hematocrit 22.3%, platelet count 200∗10^3^/uL, mean corpuscular volume 88.1 fL, reticulocyte count 3.33%, fibrinogen 601 mg/dL, haptoglobin 229 mg/dL, lactate dehydrogenase 234 U/L, prothrombin time (PT) 13.7 s, activated partial thromboplastin time (aPTT) 65.6 s (normal: 24.7 s–39.8 s), blood urea nitrogen (BUN) 29 mg/dL, creatinine 3.41 mg/dL, corrected calcium 9.3 mg/dL, aspartate aminotransferase (AST) 34 U/L, alanine aminotransferase (ALT) 15 U/L, alkaline phosphatase 129 U/L, albumin 2.8 g/dL, total bilirubin 0.70 mg/dL, complement component 3 (C3) 106 mg/dL, complement component 4 (C4) 22 mg/dL, and cluster of differentiation 4 (CD4) count 318/uL. Urinalysis with automicroscopy showed blood without the presence of red cell casts. Computed tomography angiogram (CTA) of the abdominal arteries without the use of intravenous contrast revealed large bilateral retroperitoneal hematomas that extended inferiorly to the inguinal canal and a right thigh hematoma (Figure 1). The patient was admitted to the intensive care unit and over the next two days received a total of 10 units of packed red blood cells (pRBCs), 4 units of fresh frozen plasma (FFP), 1 unit of cryoprecipitate, and 5 grams of aminocaproic acid. Further laboratory tests revealed prostate-specific antigen 0.33 ng/mL, normal immunoglobulin levels, trace monoclonal Kappa IgG 0.43 g/dL (PTT mixing 1 : 1 dilution 44 s, 2 hours: 66 s, 50 : 50 mix at 2 h: 58.3 s), thrombin time 15.5 s (normal: 14.0 s–19.1 s), PTT lupus anticoagulant: 69 s (normal: <40 s), dilute Russell's viper venom time (dRVVT) 49 s (normal: <48 s), positive hexagonal phospholipid confirmatory test, factor II activity 95 (normal: 70–150%), factor VIII activity <1% (normal: 50–180%), factor IX activity 112% (normal: 60–160%), factor XI 56% (normal: 65–150%,) factor XII activity 49% (normal: 50–150%), and factor VIII inhibitor 230.40 Bethesda units (Table 1). The diagnosis of acquired factor VIII was made, and patient was started on solumedrol 80 mg intravenously every eight hours, cyclophosphamide 50 mg orally daily, and recombinant coagulation factor VIIa 2000 mcg intravenously three times a day. The patient initially failed to respond to treatment, requiring 30 transfusions of packed red blood cells. However, after five weeks of therapy, most of which were spent in the intensive care unit, the patient's hemoglobin stabilized at 12.0 g/dL with a PTT of 43.4 s and a factor VIII activity of 23% (Table 2), with hematoma stability seen on repeat computed tomography of the abdomen and pelvis (Figure 2). He was sent home with outpatient follow-up on cyclophosphamide and prednisone.

## 3. Discussion

Our presentation of a severe case of a very rare condition brings to light many interesting issues related to the pathogenesis, presentation, diagnosis, and treatment of acquired FVIII.

### 3.1. Pathophysiology/Disease Associations

FVIII inhibitors are known to bind to the highly antigenic C2 and A2 domains on FVIII which in turn leads to reduced procoagulant activity [34–36]. The reason for the development of these inhibitors in certain individuals is poorly understood. Mahendra et al. theorized that the presence of certain gene polymorphisms or autoreactive CD4+ T lymphocytes accounts for individual variation [37].

Our case is unique in that our patient had many different possible causes for his acquired FVIII inhibitor. At the time of admission, our patient was taking many different medications, but none of which were started over the previous month, and none of which have been associated with FVIII inhibitors. Drug induction of inhibitors, accounting for 5–10%, has been associated with penicillin, sulfamides, chloramphenicol, methyldopa, depot thioxanthene, phenytoin, interferon, and fludarabine [5, 23, 38]. Our patient was found to have a lupus anticoagulant (LA), in the absence of obvious systemic lupus erythematosus (SLE) or rheumatoid arthritis (RA). Both of these disease entities, in addition to other autoimmune conditions, such as Sjogrens' syndrome (SS), Goodpasture's syndrome, myasthenia gravis (MG), Graves disease, and autoimmune hemolytic anemia, have been shown to induce inhibitors [3, 13–15]. Although our patient had a normal factor II (prothrombin) level, the presence of LA has been associated with antibodies against prothrombin, in what is known as lupus anticoagulant hyperprothrombinemia syndrome (LAC-HPS; [39–41]). 

Our patient was found to have a monoclonal gammopathy of unknown significance (MGUS) with 0.43 grams of IgG Kappa. It is unclear whether this patient had an associated lymphoproliferative disorder, as a bone marrow biopsy was not performed, and whether or not this was related to the patient's HCV [42]. There was no evidence of recurrent lymphoma in the multiple CT scans performed during his hospital stay. It is also unclear whether this finding was incidental or was playing a larger role in the production of autoantibodies. Several reviews have shown that both solid tumors and hematologic malignancies can cause this phenomenon, with lung and prostate adenocarcinoma and low grade lymphoproliferative diseases being the most common culprits [17–22]. Although there have been several case reports describing patients with HCV who developed acquired FVIII inhibitors, most of these patients were undergoing treatment with interferon, a known immunomodulatory agent [24–28]. Schreiber and Bräu proposed that the presence of autoantibodies to FVIII in HCV was in fact extrahepatic autoimmune manifestations similar to cryoglobulinemia and hepatitis-induced thrombocytopenia [24]. There have been several case reports associating inhibitors with HIV, but these were all seen in patients receiving HAART and were attributed to IRIS [28–32]. Migliore et al. hypothesized that the predilection for antibody production in HIV-infected patients was due to the combination of T-cell imbalance, dysregulation of cytokine and antibody formation, and the dysregulation of plasma cells [28].

### 3.2. Clinical Features/Diagnosis

As was seen in our patient, the clinical presentation of acquired FVIII is often life threatening and usually involves large, rapidly expanding hematomas, uncontrolled gastrointestinal bleeding, and/or hematuria. Hemarthrosis, as seen with the inherited FVIII deficiency, is rarely seen with an acquired FVIII inhibitor [5]. A classic presentation, in addition to an elevated aPTT, is often diagnostic. However, several other conditions can give an elevated aPTT in the setting of bleeding, including deficiencies or inhibitors of factors VIII, IX, or XI, Von Willebrand disease (VWD), and the iatrogenic use of heparin. The presence of heparin can be assumed with a prolonged thrombin time and a normal reptilase time. In a mixing study, a patient's plasma is mixed with pooled normal plasma, and the aPTT is measured immediately and two hours afterward. Correction of the aPTT suggests factor deficiency or VWD, whereas an unchanged or minimally corrected aPTT represents the presence of an inhibitor. The Bethesda assay has great utility, as it not only establishes the diagnosis of acquired FVIII inhibitor but also quantifies the titer [43]. In the assay, serial dilutions of patient plasma are incubated in normal patient plasma for two hours; the stronger the inhibitor, the greater the dilution required to allow for factor VIII activity.

### 3.3. Treatment

Control of bleeding and the elimination of the inhibitor are the primary goals of treatment. The initial treatment is primarily based on the severity of the bleeding and the titer of the inhibitor [38, 44, 45]. Non-life-threatening bleeding with low inhibitor titers can be treated with desmopressin (DDAVP) or high dose human factor VIII concentrate, whereas more substantial bleeding and higher inhibitor titers call for more aggressive measures, including bypassing agents such as activated prothrombin complex concentrate FVIII bypassing agent (FEIBA) and human recombinant factor VIIa (rFVIIa) [46–49]. FEIBA has shown complete response rates of 76% with severe bleeds and 100% with moderate bleeds [47], whereas rFVIIa has shown an overall efficacy of 95% in the first line setting and 80% as salvage therapy [48, 49]. Although there are no randomized clinical trials demonstrating superiority of a particular regimen, immunosuppressive therapy is the cornerstone for the elimination of factor inhibitors. In a large registry, the most commonly employed regimens were glucocorticoids (G), glucocorticoids plus cyclophosphamide (GC), and glucocorticoids plus rituximab (GR), with complete response (CR) rates of 48%, 70%, and 59%, respectively, with a significantly shorter time to a negative inhibitor and normal FVIII level in the GC group but no difference in overall outcomes [7]. In a large literature review, the CR for GC was significantly greater than for G at 78% and 70%, respectively [50]. Although rituximab was introduced as a potential novel agent for the treatment of acquired FVIII, only anecdotal studies have demonstrated efficacy [7]. Rituximab has therefore been relegated as second-line therapy in such cases. Intravenous immune globulin (IVIG) demonstrated activity in a select group of patients, but responses were highly variable [13, 45, 51]. In treatment-resistant acquired FVIII inhibitors, there was anecdotal evidence for the use of cyclosporine, cladribine, vincristine, and extracorporeal plasmapheresis [52–59]. 

### 3.4. Natural History/Prognosis

Although most patients with acquired FVIII are treated with immunosuppressive drugs, there are a significant number of patients who recover spontaneously. Studies cite a spontaneous recovery rate of 36% and 31% at an average duration of 14 and 31 months, respectively [3, 60]. The patient we presented had a long protracted hospital course, requiring numerous transfusions of blood products, before he began clearing the inhibitor and restoring factor VIII activity. His course was not unusual, as patients with low antibody titers (<5 Bethesda units) tend to have remissions within months, whereas those with higher titers may have antibody persistence for years. Low antibody titers and those with pregnancy-associated FVIII inhibitors appear to respond best to treatment and have the lowest relapse rates [2, 42]. The overall relapse rate is estimated at 20%, with 70% of these patients achieving a second remission. The overall prognosis varies, with mortality rates ranging from 8 to 22%, with fatal bleeding (3.2%) being very uncommon [6]. Evidence shows that GC, especially if given to the elderly, is associated with significant adverse effects in 40% of patients, most commonly infection and neutropenia. Meanwhile, G (25%) and GR (37%) are associated with less adverse effects, most commonly infection and diabetes, respectively [7]. The survival rate at 8 months for acquired FVIII inhibitor secondary to all causes is 69%, with those secondary to malignancy having the worst outcomes [7].

## 4. Conclusion

Our case of a very rare condition highlights the importance of recognizing and understanding the diagnosis of acquired FVIII inhibitor. Laboratory research and clinical data on the role of new agents are needed in order to better characterize disease pathogenesis, disease associations, genetic markers, and optimal disease management. The hope is to one day better identify patients who are at increased risk for the disorder and then personalize treatment regimens in order to improve disease morbidity and mortality.

## Figures and Tables

**Table 1:** Comparison between a meta-analysis by Delgado et al. [33], a case series by Collins et al. [2], and our patient**. **

Characteristics	Delgado et al. [33] (2003)	Collins et al. [2] (2007)	Our patient (2013)
Number of patients	249	154	1
Age category (median)	64 (range, 8–93)	78 (range, 2–98)	57
Sex	Female: 55%	Female: 57.38	Male
Underlying diagnosis	Malignancy Postpartum status Autoimmune disorders	None: 63.3% Autoimmune or collagen vascular disease: 16.7% Malignancy: 14.7	HCV, HIV, LA, MGUS
FVIII level category at diagnosis (median)	2 IU/dL (range, 0–30)	4 IU/dL (range, <1–12 IU/dL)	<1 IU/dL
Inhibitor titer at diagnosis (median)	10.0 BU/mL (range, 0.9–32,000)	7.2 BU/mL (range, 1.4–219 BU/mL)	230.4 BU/mL

**Table 2:** Laboratory values and treatment regimen used during patient's hospital course.

	Admission	Day 7	Day 21	Day 35
Hemoglobin (g/dL)	7.2	8.6	10.0	11.4
Hematocrit	22.3	26.3	31.6	37.9
Platelet count (/uL)	200∗10^3^	204∗10^3^	130∗10^3^	160∗10^3^
PTT (s)	65.6	62.4	63.5	44.3
Factor VIII activity (%)	<0.1	3	14	23
Total pRBCs used	—	15	25	30
Treatment regimen	—	(1) Solumedrol 80 mg IV q 8 hrs (2) Cyclophosphamide 50 mg PO daily (3) Recombinant coagulation factor VIIa 2000 mcg IV BID	(1) Solumedrol 80 mg IV q 8 hrs (2) Cyclophosphamide 50 mg PO daily (3) Recombinant coagulation factor VIIa 1000 mcg IV TID	(1) Solumedrol 80 mg IV q 8 hrs (2) Cyclophosphamide 50 mg PO daily

## References

[1] Collins P, Macartney N, Davies R, Lees S, Giddings J, Majer R (2004). A population based, unselected, consecutive cohort of patie with acquired haemophilia A. *British Journal of Haematology*.

[2] Collins PW, Hirsch S, Baglin TP (2007). Acquired hemophilia A in the United Kingdom: a 2-year national surveillance study by the United Kingdom Haemophilia Centre Doctors’ organisation. *Blood*.

[3] Green D, Lechner K (1981). A survey of 215 non-hemophilic patients with inhibitors to factor VIII. *Thrombosis and Haemostasis*.

[4] Hauser I, Schneider B, Lechner K (1995). Post-partum factor VIII inhibitors: a review of the literature with special reference to the value of steroid and immunosuppressive treatment. *Thrombosis and Haemostasis*.

[5] Franchini M, Gandini G, Di Paolantonio T, Mariani G (2005). Acquired hemophilia A: a concise review. *The American Journal of Hematology*.

[6] Knoebl P, Marco P, Baudo F (2012). Demographic and clinical data in acquired hemophilia A: results from the European Acquired Haemophilia Registry (EACH2). *Journal of Thrombosis and Haemostasis*.

[7] Collins P, Baudo F, Knoebl P (2012). Immunosuppression for acquired hemophilia A: results from the European Acquired Haemophilia Registry (EACH2). *Blood*.

[8] Green D, Schuette PT, Wallace WH (1980). Factor VIII antibodies in rheumatoid arthritis. Effect of cyclophosphamide. *Archives of Internal Medicine*.

[9] Soriano RMG, Matthews JM, Guerado-Parra E (1987). Acquired haemophilia and rheumatoid arthritis. *British Journal of Rheumatology*.

[10] Lafferty TE, Smith JB, Schuster SJ, DeHoratius RJ (1997). Treatment of acquired factor VIII inhibitor using intravenous immunoglobulin in two patients with systemic lupus erythematosus. *Arthritis and Rheumatism*.

[11] Ballard HS, Nyamuswa G (1993). Life-threatening haemorrhage in a patient with rheumatoid arthritis and a lupus anticoagulant coexisting with acquired autoantibodies against factor VIII. *British Journal of Rheumatology*.

[12] Hauser I, Lechner K (1999). Solid tumors and factor VIII antibodies. *Thrombosis and Haemostasis*.

[13] Tiplady CW, Hamilton PJ, Galloway MJ (2000). Acquired haemophilia complicating the remission of a patient with high grade non-Hodgkin’s lymphoma treated by fludarabine. *Clinical and Laboratory Haematology*.

[14] English KE, Brien WF, Howson-Jan K, Kovacs MJ (2000). Acquired factor VIII inhibitor in a patient with chronic myelogenous leukemia receiving interferon-alfa therapy. *Annals of Pharmacotherapy*.

[15] Komminoth A, Dufour P, Bergerat JP, Wiesel ML, Falkenrodt A, Oberling F (1992). Hairy cell leukemia and factor VIII inhibitor: a case report. *Nouvelle Revue Francaise d’Hematologie*.

[16] Bossi P, Cabane J, Ninet J (1998). Acquired hemophilia due to factor VIII inhibitors in 34 patients. *The American Journal of Medicine*.

[17] Yee TT, Pasi KJ, Lilley PA, Lee CA (1999). Factor VIII inhibitors in haemophiliacs: a single-centre experience over 34 years, 1964-97. *British Journal of Haematology*.

[18] Franchini M (2006). Postpartum acquired factor VIII inhibitors. *The American Journal of Hematology*.

[19] Tengborn L, Baudo F, Huth-Kühne A (2012). Pregnancy-associated acquired haemophilia A: results from the European Acquired Haemophilia (EACH2) registry. *BJOG*.

[20] Delyon J, Mateus C, Lambert FT (2011). Hemophilia A induced by ipilimumab. *The New England Journal of Medicine*.

[21] Sallah S, Wan JY (2001). Inhibitors against factor VIII associated with the use of interferon-alpha and fludarabine. *Thrombosis and Haemostasis*.

[22] Sborov DW, Rodgers GM (2012). Acquired hemophilia A: a current review of autoantibody disease. *Clinical Advances in Hematology and Oncology*.

[23] Paul S, Javed U, Tevendale R, Lanford J, Liu R (2007). Acquired factor VIII inhibitor in an HIV-infected patient after treatment with pegylated interferon-*α* 2a and ribavirin. *AIDS*.

[24] Schreiber ZA, Bräu N (2005). Acquired factor VIII inhibitor in patients with hepatitis C virus infection and the role of interferon-*α*: a case report. *The American Journal of Hematology*.

[25] Herman C, Boggio L, Green D (2005). Factor VIII inhibitor associated with peginterferon. *Haemophilia*.

[26] Castenskiold EC, Colvin BT, Kelsey SM (1994). Acquired factor VIII inhibitor associated with chronic interferon-alpha therapy in a patient with haemophilia A. *British Journal of Haematology*.

[27] Dentale N, Fulgaro C, Guerra L, Fasulo G, Mazzetti M, Fabbri C (1997). Acquisition of factor VIII inhibitor after acute hepatitis C virus infection. *Blood*.

[28] Migliore E, Allione A, Dutto L (2009). Acquired factor VIII inhibitor in patient infected with HIV: a casual association or a prone immunological setting?. *Haemophilia*.

[29] Lake DF, Schluter SF, Wang E, Bernstein RM, Edmundson AB, Marchalonis JJ (1994). Autoantibodies to the *α*/*β* T-cell receptors in human immunodeficiency virus infection: dysregulation and mimicry. *Proceedings of the National Academy of Sciences of the United States of America*.

[30] Gringeri A, Santagostino E, Mannucci PM (1994). A randomized, placebo-controlled, blind anti-AIDS clinical trial: safety and immunogenicity of a specific anti-IFN*α* immunization. *Journal of Acquired Immune Deficiency Syndromes*.

[31] Indraccolo S, Mion M, Zamarchi R (1993). B cell activation and human immunodeficiency virus infection. V. Phenotypic and functional alterations in CD5+ and CD5- B cell subsets. *Journal of Clinical Immunology*.

[32] Werwitzke S, Tiede A, Stoll M, von Depka M (2004). Immune reconstitution inflammatory syndrome (IRIS) as a cause for inhibitor development in hemophilia. *Journal of Thrombosis and Haemostasis*.

[33] Delgado J, Jimenez-Yuste V, Hernandez-Navarro F, Villar A (2003). Acquired haemophilia: review and meta-analysis focused on therapy and prognostic factors. *British Journal of Haematology*.

[34] Prescott R, Nakai H, Saenko EL (1997). The inhibitor antibody response is more complex in hemophilia a patients than in most nonhemophiliacs with factor VIII autoantibodies. *Blood*.

[35] Arai M, Scandella D, Hoyer LW (1989). Molecular basis of factor VIII inhibition by human antibodies. Antibodies that bind to the factor VIII light chan prevent the interaction of factor VIII with phospholipid. *Journal of Clinical Investigation*.

[36] Scandella D, Gilbert GE, Shima M (1995). Some factor VIII inhibitor antibodies recognize a common epitope corresponding to C2 domain amino acids 2248 through 2312, which overlap a phospholipid-binding site. *Blood*.

[37] Mahendra A, Padiolleau-Lefevre S, Kaveri SV, Lacroix-Desmazes S (2012). Do proteolytic antibodies complete the panoply of the autoimmune response in acquired haemophilia A?. *British Journal of Haematology*.

[38] Hay CRM, Brown S, Collins PW, Keeling DM, Liesner R (2006). The diagnosis and management of factor VIII and IX inhibitors: a guideline from the United Kingdom Haemophilia Centre Doctors Organisation. *British Journal of Haematology*.

[39] Vivaldi P, Rossetti G, Galli M, Finazzi G (1997). Severe bleeeding due to acquired hypoprothrombinemia-lupus anticoagulant syndrome. Case report and review of literature. *Haematologica*.

[40] Erkan D, Bateman H, Lockshin MD (1999). Lupus anticoagulant-hypoprothrombinemia syndrome associated with systemic lupus erythematosus: report of 2 cases and review of literature. *Lupus*.

[41] Hara Y, Makita M, Ishikawa T (2013). Lupus anticoagulant hypoprothrombinemia syndrome in Bence-Jones protein kappa-type multiple myeloma patient with phosphatidylserine-dependent antiprothrombin antibody. *Annals of Hematology*.

[42] Giordano TP, Henderson L, Landgren O (2007). Risk of non-Hodgkin lymphoma and lymphoproliferative precursor diseases in US veterans with hepatitis C virus. *Journal of the American Medical Association*.

[43] Kasper CK, Aledort L, Aronson D (1975). Proceedings: a more uniform measurement of factor VIII inhibitors. *Thrombosis et Diathesis Haemorrhagica*.

[44] Collins PW (2007). Treatment of acquired hemophilia A. *Journal of Thrombosis and Haemostasis*.

[45] Collins PW, Percy CL (2010). Advances in the understanding of acquired haemophilia A: implications for clinical practice. *British Journal of Haematology*.

[46] Franchini M, Lippi G (2008). Acquired factor VIII inhibitors. *Blood*.

[47] Sallah S (2004). Treatment of acquired haemophilia with factor eight inhibitor bypassing activity. *Haemophilia*.

[48] Hay CRM, Negrier C, Ludlam CA (1997). The treatment of bleeding in acquired haemophilia with recombinant factor VIIa: a multicentre study. *Thrombosis and Haemostasis*.

[49] Baudo F, Collins P, Huth-Kühne A (2012). Management of bleeding in acquired hemophilia A: results from the European Acquired Haemophilia (EACH2) Registry. *Blood*.

[50] Collins PW (2011). Management of acquired haemophilia A. *Journal of Thrombosis and Haemostasis*.

[51] Schwartz RS, Gabriel DA, Aledort LM, Green D, Kessler CM (1995). A prospective study of treatment of acquired (autoimmune) factor VIII inhibitors with high-dose intravenous gammaglobulin. *Blood*.

[52] Schulman S, Langevitz P, Livneh A, Martinowitz U, Seligsohn U, Varon D (1996). Cyclosporine therapy for acquired factor VIII inhibitor in a patient with systemic lupus erythematosus. *Thrombosis and Haemostasis*.

[53] Brox AG, Laryea H, Pelletier M (1998). Successful treatment of acquired factor VIII inhibitors with cyclosporin. *The American Journal of Hematology*.

[54] Maclean PS, Campbell Tait R, Lowe GDO, Walker ID, McColl MD (2003). Successful elimination of factor VIII inhibitor using cyclosporin A. *British Journal of Haematology*.

[55] Petrovic M, Derom E, Baele G (2000). Cyclosporine treatment of acquired hemophilia due to factor VIII antibodies. *Haematologica*.

[56] Sallah S, Wan JY (2003). Efficacy of 2-chlorodeoxyadenosine in refractory factor VIII inhibitors in persons without hemophilia. *Blood*.

[57] Jansen M, Schmaldienst S, Banyai S (2001). Treatment of coagulation inhibitors with extracorporeal immunoadsorption (Ig-Therasorb). *British Journal of Haematology*.

[58] Freedman J, Rand ML, Russell O (2003). Immunoadsorption may provide a cost-effective approach to management of patients with inhibitors to FVIII. *Transfusion*.

[59] Zeitler H, Ulrich-Merzenich G, Hess L (2005). Treatment of acquired hemophilia by the Bonn-Malmö Protocol: documentation of an in vivo immunomodulating concept. *Blood*.

[60] Lottenberg R, Kentro TB, Kitchens CS (1987). Acquired hemophilia. A natural history study of 16 patients with factor VIII inhibitors receiving little or no therapy. *Archives of Internal Medicine*.

